# TGF*-*β expression on different suturing technique for abdominal skin wound closure in rats

**DOI:** 10.1016/j.amsu.2021.102521

**Published:** 2021-06-20

**Authors:** Imam Sofii, Ratna Sari Kalembu, Aditya Rifqi Fauzi, Firdian Makrufardi, Akhmad Makhmudi

**Affiliations:** a Digestive Surgery Division, Department of Surgery, Faculty of Medicine, Public Health and Nursing, Universitas Gadjah Mada/Dr. Sardjito Hospital, Yogyakarta, 55281, Indonesia; b Pediatric Surgery Division, Department of Surgery, Faculty of Medicine, Public Health and Nursing, Universitas Gadjah Mada/Dr. Sardjito Hospital, Yogyakarta, 55281, Indonesia

**Keywords:** Abdominal skin, Small stitch, Large stitch, Wound closure, TGF-β

## Abstract

**Background:**

The method of closing the abdominal wall, as well as, the choice of material for stitching are important aspects of efficient incision closure. Generally, transforming growth factor-beta (TGF-β) is involved in the wound healing process. Suturing procedures also play a part in the wound dehiscence occurrence. This study aimed to compare TGF-β expressions in rats after using the large stitch vs. small stitch technique for abdominal skin wound closure.

**Methods:**

A total of twenty Wistar rats (*Rattus norvegicus*) were used in this experiment. Small tissue bites of 5 mm were obtained by the small stitch group and the large stitch group received large bites of 10 mm. Abdominal skin incisions were closed by running sutures. On days 4 and 7, the animals were euthanized. For TGF-β expressions, histological parts of the tissue-embedded sutures were analyzed. With significance set at *p <* 0.05*,* two-way ANOVA showed that on days 4 and 7, the TGF-β expressions of the rats in the small stitch group were nearly identical to those in the large stitch groups.

**Results:**

After including twenty rats in this study, results showed the TGF-β expressions on days 4 and 7 in rats in the small stitch group were equivalent to those in the large stitch group. (*p* = 0.45).

**Conclusions:**

Between the small and the large stitch groups, the TGF-β expressions are similar, suggesting that the suturing methods do not have any significantly different beneficial impact on the frequency of wound dehiscence.

## Highlights

• TGF-β promoting collagen and fibronectin formation in various fibroblast cell lines.
• TGF-β expressions are similar between the small and the large stitch groups.
• Different suturing methods do not have a beneficial impact on the frequency of wound dehiscence.

## 1 Background

As a postoperative complication, wound dehiscence is frequently noticed and causes patients discomfort and prolonged stay. Several causes are known to be associated with wound dehiscence, including old age, malnutrition, anemia, hypoalbuminemia, obesity and most importantly, infection [1]. The suturing process is another related factor. Previous studies have established a link between wound dehiscence and wound suturing techniques [2,3].

The accuracy of stitching techniques is critical in avoiding wound complications in the median incision, according to a number of clinical and laboratory studies [4,5].

The occurrence of wound dehiscence is a worrying complication of postoperative wounds. One common factor causing wound dehiscence is surgical site infection. Bacterial infection can cause influx and neutrophil activation and can also increase the degradation of matrix metalloproteinase [6].

Transforming growth factor-beta (TGF-β) is known to play a role in promoting collagen and fibronectin formation in various fibroblast cell lines [7]. TGF-β is known to control cell differentiation, induce chemotaxis of inflammatory cells, and induce in vivo accumulation of extracellular matrix protein [8].

In order to avoid wound dehiscence, several studies have shown the advantages of the small stitch suturing technique over the large stitch. However, this tudy did not concentrate on the impact of suturing techniques on TGF-β expression in the prevention of wound dehiscence. Accordingly, this research aimed to compare the effects of large stitches on TGF-β expressions in rats with small stitches for abdominal skin closure in rats.

## 2 Material and methods

### 2.1 Animal models

Wistar rats (*Rattus norvegicus*) were used in an experimental sample. Twenty 170–200 g rats were selected for this study and were adapted for a week prior to the experiment. Rats that were sick, had an infection during the procedures, or died during the procedures were excluded from this study. Our research was in line with experimental animal guidelines: 3R (replacement, reduction, and refinement) and 5F (freedom of hunger and thirst, freedom from discomfort, freedom of pain, injury, or disease, freedom to fear and distress, and freedom to express natural behavior).

**Fig. 1:**
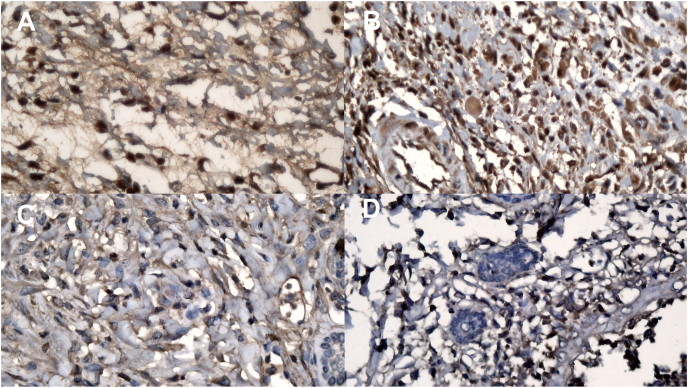
Small-stitch TGF-β expressions on: a) day 4 and b) day 7, and large-stitch expressions on: c) day 4 and d) day 7. Among groups and days, TGF-β expressions were similar (p > 0.05).

Our subjects were 20 rats divided into four groups (each with five rats): group of large stitches and euthanized on day 4 was marked as group 1, group of small stitches and euthanized on day 4 was marked as group 2, group of large stitches and euthanized on day 7 was marked as group 3, and group of small stitches and euthanized on day 4 was marked as group 4. Each rat was euthanized by decapitation. After euthanasia, samples of abdominal skin that contained the stitches were taken (1 × 0.5 cm) and stained with TGF-β using immunohistochemistry methods (Fig. 1).

All sedation procedures were performed with a 60 mg/kg intramuscular injection of ketamine (Ikapharmindo, Jakarta, Indonesia) after 1% povidone iodine aseptic-antiseptic action. Supplementary doses were used to keep the anesthesia going as required. The animals were held supine during the experiments. The incision into the rat's abdominal skin was 6 cm long, followed by a 6 cm incision into the alba line. Both of the stitches were placed in the small stitch group, 5 mm from the wound's edge, and the big stitch group, 10 mm from the wound's edge. The suture technique was done with polyvinylidene fluoride monofilament using continuous suture and suture content.

An immunohistochemical assay was used to analyze the TGF-β expression of paraffin blocks. A counter in the Adobe Reader program was used to count the number of TGF-β expressions. The researchers did not sample any different area for comparison. Dark brown cells emerged as expressions of TGF-β. These experimental procedures were performed with the prior approval of the Medical and Health Research Ethics Committee of the Faculty of Medicine, Public Health and Nursing, Universitas Gadjah Mada/Dr. Sardjito Hospital (KE/FK/0151/EC/2018). The work has been reported in line with the ARRIVE statement [9].

### 2.2 Statistical analysis

SPSS 24 version (IBM Corp., New York) was used to perform statistical analysis on the data. Following normality testing and hypothetical tests comparing each intervention group, a 2-way ANOVA was used. Comparing means with standard deviation (SD), significance was reached if *p* < 0.05.

## 3 Results

After immunohistochemical staining on tissue samples from each group, TGF-β expressions identified as dark brown cells. (Fig. 1).

Twenty rats were included in this study. We found that TGF- β expressions in the small stitch group was 48.33 ± 6.20 (mean ± SD) and 43.20 ± 7.23 (mean ± SD) on day 4 and day 7 respectively. In the large stitch group TGF- β expressions was 39.00 ± 14.82 (mean ± SD) on day 4 and 30.80 ± 9.41 (mean ± SD) on day 7. Rats in the small stitch group had TGF-β expressions similar to those in the large stitch group on days 4 and 7 (*p* = 0.45) (Table 1).

**Table 1:** The expression of TGF- β was compared between the small and large stitch groups on days 4 and 7.

	TGF-β expressions		*p*-value
	Small stitch (mean ± SD)	Large stitch (mean ± SD)	
Day 4	48.33 ± 6.20	39.00 ± 14.82	0.45*
Day 7	43.20 ± 7.23	30.80 ± 9.41	

SD, standard deviation; TGF-β, transforming growth factor beta; *p*-value was calculated using 2-way ANOVA.

## 4 Discussion

This study's results show that in the initial phase of wound healing, the small stitch technique is not better in healing abdominal skin wounds. This finding was supported by the similar TGF-β levels, which, although the values slightly differed, the difference was not statistically significant. TGF-β plays an important role in wound healing by regulating leukocyte infiltration, angiogenesis, and collagen deposition. TGF-β also plays a direct role in the proliferation and remodeling process of wound healing by facilitating collagen deposition and angiogenesis [10].

A 5-year randomized control trial study was performed by Millbourn [5] and found that surgical wound infection and incisional hernias are less common when small stitches are used. Multivariate analysis showed a doubling of the risk of infection and a four-fold increase in the risk of incisional hernia in the long-stitch community. In the large stitch group, the low amount of TGF-β can cause impaired skin wound healing. Failure of the acute inflammation process can result in poor skin wound healing. TGF-β deficiency results in compromised immunity to infection and acute inflammatory disorders following damage to tissue and infection [11].

Fortenyl [12] in a 5-year prospective randomized control trial study compared the findings of abdominal wall closure using the same thread, namely extra-long-term absorbable monofilament, based on long stitch (10 mm interval) and short stitch (5 mm interval) suture techniques and concluded that the 5 mm small stitch technique with elastic thread absorbed monofilament was stronger than the 10 mm broad stitch suture technique to avoid incision.

This study uses polyglecaprone-25 monofilament threads, which are stronger, tissue traction is less and the risk of developing infection is low. Accordingly, the probability of postoperative wound infection is also lower than that of multifilament, so monofilament threads are used more frequently [13]. The monofilament yarn is superior over multifilament in the prevention of surgical wound infection and aided by the option of polyglecaprone-25 thread, its greater biostability will indirectly prevent TGF-β deficiency. An experimental study by Lin [10] using double-color immunofluorescent or immunohistochemical analysis after 14 days showed that angiogenesis and collagen deposition were delayed in mice with TGF-β deficiency due to a decrease in the amount of angiogenic and fibrogenic growth factors. Collectively, for wound healing, angiogenesis and collagen deposition are indispensable. TGF-β deficiency results in angiogenesis and collagen deposition that is delayed.

There were some limitations to our analysis. First, the development of wound dehiscence could be caused by several variables, including wound strain, increased abdominal pressure, malnutrition, and suture content, among others. Second, the animal model in our research is not a model of wound dehiscence. Third, we used a relatively small sample and only 1-week of observation, which could limit the generalizability of our finding. Therefore, a significant disparity remains between our results and their possible implementation in clinical practice.

## 5 Conclusions

The expression of TGF- is similar in the small and large stitch classes, meaning that different suturing strategies can have little effect on the frequency of wound dehiscence. Future larger studies are necessary to probe any potential correlation short and long stitch suturing with dehiscence occurrence.

## Provenance and peer review

Not commissioned, externally peer-reviewed.

## Funding

This research did not receive any specific grant from funding agencies in the public, commercial, or not-for-profit sectors.

## Ethical approval

Not applicable.

## Consent

Not applicable.

## Author contributions

Imam Sofii, Ratna Sari Kalembu conceived the study and approved the final draft. Aditya Rifqi Fauzi and Firdian Makrufardi drafted the manuscript. Akhmad Makhmudi critically revised the manuscript for important intellectual content. All authors read and approved the final draft. All authors facilitated all project-related tasks.

## Trial registry number

Not applicable.

## Guarantor

Imam Sofii.

## Declaration of competing interest

No potential conflict of interest relevant to this article was reported.

## Abbreviations

TGF-β: Transforming growth factor beta
SD: Standard deviation
POD: Postoperative day
